# Genetics of Insulin Resistance and the Metabolic Syndrome

**DOI:** 10.1007/s11886-016-0755-4

**Published:** 2016-06-16

**Authors:** Audrey E. Brown, Mark Walker

**Affiliations:** Institute of Cellular Medicine, William Leech Building, Medical School, Newcastle University, Newcastle, NE2 4HH UK

**Keywords:** Insulin resistance, Metabolic syndrome, Common genetic variation, Genetic risk score, Rare variants

## Abstract

Insulin resistance and the metabolic syndrome are complex metabolic traits and key risk factors for the development of cardiovascular disease. They result from the interplay of environmental and genetic factors but the full extent of the genetic background to these conditions remains incomplete. Large-scale genome-wide association studies have helped advance the identification of common genetic variation associated with insulin resistance and the metabolic syndrome, and more recently, exome sequencing has allowed the identification of rare variants associated with the pathogenesis of these conditions. Many variants associated with insulin resistance are directly involved in glucose metabolism; however, functional studies are required to assess the contribution of other variants to the development of insulin resistance. Many genetic variants involved in the pathogenesis of the metabolic syndrome are associated with lipid metabolism.

## Introduction

Both insulin resistance and the metabolic syndrome are powerful risk factors for the development of cardiovascular disease and type 2 diabetes (T2D). Insulin resistance can be defined as the inability of insulin to stimulate glucose disposal, and when an insulin-resistant individual is unable to secrete sufficient insulin to overcome this defect, T2D develops. The metabolic syndrome comprises a number of risk factors which occur together more often than by chance alone. A joint scientific statement issued in 2009 by the World Health Organisation (WHO), International Diabetes Federation (IDF), American Heart Association (AHA) and National Heart, Lung and Blood Institute (NHLBI) identified the metabolic syndrome as comprising hypertension, dyslipidaemia (raised triglyceride and low high-density lipoprotein cholesterol levels), hyperglycaemia and central obesity [1].

The prevalence of insulin resistance and the metabolic syndrome is increasing, particularly in developing countries and in younger populations with estimates of prevalence ranging from 20 to 40 % in different populations [2–4]. While there are strong lifestyle determinants for the development of both insulin resistance and the metabolic syndrome, it is increasingly clear that an individual’s risk of developing insulin resistance and aspects of the metabolic syndrome are also determined by genetic factors. Early familial genetic studies provided strong evidence for a genetic basis for both insulin resistance and the individual components of the metabolic syndrome [5–11], and since then, genome-wide association studies (GWAS) and, more recently, next-generation sequencing, have allowed for the identification of both common (defined by a minor allele frequency (MAF) >5 %) and rare (MAF < 0.5 %) genetic variants linked with these disease-associated traits. Associations of common genetic variation with a particular trait are generally defined with a genome-wide significance level of *p* < 5 × 10^−8^. The scope of this review is to summarise some of the genome-wide association studies (GWAS) studies and meta-analyses performed in relation to insulin resistance and the metabolic syndrome.

## Common Genetic Variation in Insulin Resistance

Since 2007, genome-wide association studies (GWAS) have identified approximately 88 loci associated with risk of developing T2D [12]. The vast majority of the loci related to T2D are primarily associated with insulin secretion and β-cell function with far fewer variants apparently influencing insulin resistance. There are a number of reasons for this, not the least that insulin resistance is strongly linked to obesity, and dissecting out the role of common genetic variation in insulin resistance in the absence of obesity has been problematic. There has also been a lack of large cohorts with reliable measures of insulin sensitivity. Indeed, many measures examine whole body insulin sensitivity and do not measure insulin sensitivity at a tissue/organ level. Table 1 lists the loci and nearby genes associated with insulin resistance identified by linkage analysis, candidate gene studies and GWAS.

**Table 1:** Variants associated with insulin resistance identified by candidate gene association studies, linkage analysis and GWAS

SNP	Nearby gene	Chromosome	Ref
rs13081389	*PPARG*	3	[27, 28]
rs972283	*KLF14*	7	[29]
rs2943641	*IRS1*	2	[31]
rs780094	*GCKR*	2	[17]
rs8050136	*FTO*	16	[29, 36, 76]
rs7903146	*TCF7L2*	10	[37, 38]
rs1208	*NAT2*	8	[13]
rs6723108, rs998451	*TMEM163*	2 2	[43]
rs35767	*IGF1*	12	[17]
rs12970134	*MC4R*	18	[46, 77]
rs17046216	*SC4MOL*	4	[48]
rs7077836	*TCERG1L*	10	[48]
rs702634 rs4311394	*ARL15*	12	[49, 78]

The GENESIS consortium is the only group to date that has published a GWAS of whole-body insulin sensitivity measured during hyperinsulinaemia (clamp/insulin suppression test) in a modest number of subjects (2764 with replication in an additional 2860 individuals) from four cohorts. The consortium found that variation in *NAT2* was associated with insulin resistance although this did not reach formal genome-wide significance (rs1208 was associated with decreased insulin sensitivity with *p* = 9.81 × 10^−7^ after adjusting for BMI). The rs1208 loci was also associated with fasting glucose, triglycerides and total cholesterol and with an increase in coronary heart disease risk. A functional relationship between *NAT2* and insulin sensitivity was confirmed in mouse 3T3-L1 adipocytes where NAT1 knockdown (the mouse orthologue of human NAT2) decreased insulin-stimulated glucose uptake and in a mouse NAT1 knockout model which showed decreased insulin sensitivity [13]. *NAT2* is involved in acetylation and influences sensitivity to certain drugs such as isoniazid, although the endogenous substrates remain to be defined (reviewed in [14]). GWAS such as this are challenging to perform in large cohorts as the protocols involved in measuring whole-body insulin sensitivity are time-consuming, labour-intensive and, as such, expensive to perform.

The hyperinsulinaemic-euglycaemic clamp is considered the gold standard method for assessing insulin sensitivity (defined for these purposes as the ability of insulin to stimulate glucose uptake) but it is a long protocol and complex to perform. Simpler measures of insulin sensitivity such as fasting insulin concentrations and leptin/adiponectin ratios [15] are often used as a surrogate marker for insulin sensitivity. Fasting insulin and glucose levels can also be used to calculate the homeostasis model assessment (HOMA). HOMA-IR is used as a measure of insulin resistance which has the advantage of being simple to obtain but is not directly comparable to the clamp measure [16].

In an effort to identify new variants associated with insulin resistance, large-scale meta-analyses of GWAS have been performed using multiple cohorts which have measures of insulin sensitivity, processing and secretion. This approach has confirmed associations of many loci with specific glycaemic traits as well as identifying new loci. The Meta-Analyses of Glucose and Insulin-related traits Consortium (MAGIC) performed meta-analyses on GWAS from 21 cohorts of non-diabetic individuals including 46,186 individuals with measures of fasting glucose and 38,238 with measures of fasting insulin and HOMA-IR as an index of insulin resistance. Twenty-five SNPs were followed up in a further 76,558 individuals with this approach identifying 16 loci associated with fasting glucose and two with fasting insulin. This study confirmed loci near *GCKR* and a newly identified loci near *IGF1* as being associated with insulin resistance [17] with these findings being replicated in a further 14 cohorts comprising 29,084 non-diabetic individuals with detailed measures of fasting proinsulin, insulin secretion and sensitivity [18]. This latter study has recently been expanded by Dimas and colleagues who examined the effect of 37 T2D risk loci on measures of insulin processing, secretion, sensitivity and clearance from up to 58,614 non-diabetic individuals with basal measures and 17,327 individuals with dynamic measures of glycaemic traits. They found that the risk loci could be subdivided into five clusters including one cluster with four loci, *PPARγ*, *KLF14*, *IRS1* and *GCKR*, associated with insulin resistance [19].

More recently, alternate approaches to identifying variants associated with insulin resistance have been developed. Manning and colleagues have developed a joint meta-analysis (JMA) approach to identify SNPs significantly associated with either fasting glucose and/or fasting insulin while both adjusting for BMI and allowing for interaction with BMI [20]. This analysis method can increase the power to detect genetic associations where interaction with the environment is unknown [21, 22]. This approach identified six loci, including five new variants, as being associated with fasting insulin levels (*IRS1*, *COBLL1*-*GRB14*, *PPP1R3B*, *PDGFC*, *UHRF1BP1* and *LYPLAL1*). Scott and colleagues used the Metabochip, a custom genotyping array containing 196,725 SNPs chosen due to their associations with a variety of cardiometabolic traits including type 2 diabetes, BMI and fasting glucose [23] and large-scale meta-analyses of up to 133,010 individuals to identify 17 loci significantly associated with fasting insulin. These loci included those previously identified by Manning, genes which have previously been associated with other metabolic traits (*TCF7L2*, *PPARG*, *FTO*, *RSPO3*, *ANKRD55*-*MAP3K1* and *ARL15*) and newly identified loci (*HIP1*, *TET2*, *YSK4*, *PEPD* and *FAM13A*) [24]. These loci have been used in two further studies to generate an insulin resistance genetic risk score to examine the relationship between variants associated with fasting insulin and an individual’s risk of developing insulin resistance and T2D [25•, 26•]. Scott and colleagues generated an insulin resistance risk score from ten variants which were also specifically associated with lower HDL and higher triglycerides (*IRS1*, *GRB14*, *ARL15*, *PPARG*, *PEPD*, *ANKRD55*-*MAP3K1*, *PDGFC*, *LYPLAL1*, *RSPO3* and *FAM13A1*) while Yaghootkar and colleagues selected 19 variants to generate their insulin resistance risk score. These studies found that the insulin resistance genetic risk score was associated with decreased insulin sensitivity and lower BMI [25•]. Yaghootkar and colleagues found that a cluster of 11 risk variants were associated with increased triglycerides and lower HDL-cholesterol along with a lower BMI, while individuals with 17 or more of these risk variants were slimmer but were at higher risk of developing T2D, coronary artery disease and hypertension compared with those individuals carrying nine or less of these insulin resistance risk variants [26•]. Together, these studies highlight that insulin resistance is not always associated with obesity and can occur in the absence of a high BMI.

## Genetic Loci Associated with Insulin Resistance

Many of the loci that have been identified as being associated with risk of developing insulin resistance harbour genes nearby that are good biological candidates for association with measures of insulin sensitivity. The *PPARγ* (peroxisome proliferator-activated receptor gamma) variant Pro12Ala was one of the first genetic variants to be identified as being reproducibly associated with a decreased risk of developing T2D [27, 28]. *PPARγ* is a nuclear receptor involved in the transcription of genes involved in fatty acid and energy metabolism, and PPARy agonists are currently used in T2D treatment. rs972283 near *KLF14* (kruppel-like factor 14) was identified through large-scale association analysis of a European cohort [29] associated with reduced insulin action, and KLF14 gene and protein expression have recently been shown to be significantly reduced in both adipose tissue and muscle from T2D patients [30]. *IRS1* (insulin receptor substrate 1) is a key component of the insulin signalling pathway initiating the activation of PI3K in response to insulin. The C allele at rs2943641 adjacent to *IRS1* was identified as being associated with insulin resistance and hyperinsulinaemia in a European population. Functional studies showed that the risk allele was associated with both lower basal IRS1 protein levels and reduced PI3K-activity during insulin infusion indicating a causative role for the variant on disease risk [31]. The SNP, rs2943650, near *IRS1* has also been associated with a lower body fat percentage, increased triglycerides and insulin resistance, and lower HDL-cholesterol [32]. *GCKR* (glucokinase regulator) encodes glucokinase regulatory protein (GKRP) which binds to, and inhibits the activity of glucokinase, a key enzyme in regulating hepatic glucose disposal and storage [33].

Variants within the first intron of *FTO* were the first to be robustly linked with body mass index [34, 35]. Further studies found significant associations of the variants with obesity-related traits and also with fasting insulin and insulin sensitivity [36]. Variants within *TCF7L2* (transcription factor 7-like 2) show the strongest and most consistent association with risk of developing T2D of any gene variants identified so far. The risk variant has been associated with both impaired beta cell function and insulin resistance [37–39]. Subsequent functional studies have focussed on the role of *TCF7L2* in beta cell function [40, 41]. However, there is now increasing evidence for a role for *TCF7L2* in insulin-dependent tissues [42]. A variant in *NAT2* (N-acetyltransferase 2) was recently found to be the top signal in four European cohorts of non-diabetic individuals who underwent genome-wide genotyping as well as a direct measure of insulin sensitivity [13]. Functional analysis of the mouse orthologue, *NAT1*, demonstrated that NAT1 knockout mice had impaired insulin sensitivity in vivo.

Two loci near *TMEM163* (transmembrane protein 163) were found to be associated with both reduced plasma insulin and HOMA-IR in a GWAS of a cohort with Indian ancestry [43]. *IGF1* (insulin-like growth factor 1) is similar to insulin in function and regulates growth and development. Low plasma levels of IGF-1 have previously been associated with a reduction in insulin sensitivity [44], and analysis of the rs35767 polymorphism indicates that carriers of the G allele have lower circulating levels of IGF-1 compared to A allele carriers [45]. *MC4R* (melanocortin 4 receptor) was identified by GWAS of a UK cohort of Indian-Asian and European ancestry as being associated with both insulin resistance, as measured by HOMA-IR and waist circumference, with higher risk-allele frequencies being found in the Indian-Asian cohort [46]. Variants within *MC4R* have also been associated with BMI [47]. GWAS of an African-American cohort identified the SNP rs7077836 near *TCERG1L* (transcription elongation regulator 1-like) and rs17046216 in *SC4MOL* (sterol-C4-methyl oxidase-like) as being associated with both fasting insulin and HOMA-IR, a finding also replicated in a West African cohort [48]. *ARL15* (ADP ribosylation factor like GTPase 15) belongs to a family of intracellular vesicle trafficking although its exact function is still unknown. Variants within *ARL15* are associated with decreased adiponectin levels and nominally associated with risk of T2D, coronary heart disease and insulin resistance as measured by HOMA-IR [49]. Adiponectin is an adipokine involved in improving insulin sensitivity (reviewed in [50]). A recent study using a novel adiponectin receptor agonist suggests that activation of the adiponectin pathway directly improves insulin sensitivity [51].

Some of the novel loci are of unknown function but others, such as *PPP1R3B* (protein phosphatase 1 regulatory subunit 3B), are thought to play a role in skeletal muscle glycogen synthesis [52] while *GRB14* (growth factor receptor bound protein 14) interacts with receptor tyrosine kinases such as the insulin and insulin-like growth factor receptors [53].

## Common Genetic Variation in Metabolic Syndrome

The search for genetic determinants of the metabolic syndrome has largely taken two approaches, either examining the metabolic syndrome as a whole or as pairs of traits, or analysing associations with the individual components of the metabolic syndrome. Before GWAS, candidate gene association studies and linkage studies reported a number of potential genes associated with aspects of the metabolic syndrome but many of these findings were not replicated. Most GWAS to date have examined associations of variants with the individual components of the metabolic syndrome with at least 56 loci being reproducibly associated with obesity, 157 with lipids and over 90 loci associated with hypertension as well as the numerous loci associated with T2D (reviewed in [54]). It is beyond the scope of this review to discuss GWAS associated with the individual metabolic syndrome components. Instead, we focus on summarizing GWAS involving the examination of the metabolic syndrome as an entity in itself. Table 2 summarizes the variants identified in such studies.

**Table 2:** Common genetic variation associated with the metabolic syndrome

SNP	Nearby gene	Chromosome	Ref
rs295 rs10790162 rs2075290 rs2266788 rs173539	*LPL* *BUD13* *ZNF259* *APOA5* *CETP*	8 11 11 11 16	[56]
rs4420638 rs11065987 rs753381 rs1260326 rs579060 rs301 rs7979473 rs9923233 rs10401969	*APOC1* *BRAP* *PLCG1* *GCKR* *ABCB11* *LPL* *HNF1A* *FTO* *SUGP1*	19 12 20 2 2 8 12 16 19	[57]
rs964184	*APOA1*/*C3*/*A4*/*A5*	11	[58]
rs11216126 rs180349	*BUD13* *BUD13*	11 11	[59]
rs12721054	*APOC1*	19	[60]
rs73989312 rs73989319 rs7964157 rs77244975 rs76822696 rs16912410 rs62526240 rs55752635 rs146816516	*CA10* *CA10* *KSR2* *CTNNA3* *RALYL* *RALYL* *RALYL* *RALYL* *MBNL1*	17 17 12 10 8 8 8 8 3	[61]

The first GWAS examining common genetic variation in metabolic syndrome was performed on a population of Indian-Asian men who have a higher prevalence of metabolic syndrome compared to the European population. A total of approximately 317,000 SNPs were genotyped in 2700 individuals in stage 1 with the top 1500 SNPs followed up in 2300 individuals in stage 2. No common genetic basis for metabolic syndrome was identified in this study [55]. GWAS of a European population examined the association of approximately 2.5 million SNPs with either metabolic syndrome as a whole, or with pairs of traits in 22,161 individuals. Variants near *BUD13*, *ZNF259*, *APOA5*, *LPL* and *CETP* were found to be associated with metabolic syndrome, and a further 27 variants were associated with combinations of pairs of traits [56]. Avery and colleagues adopted a different approach to examining the metabolic syndrome involving the clustering of metabolic traits into six phenotype domains encompassing 19 quantitative traits. Using data from 19,486 European and 6287 African-American individuals, they identified three new loci associated with more than one phenotype domain, *APOC1*, *BRAP* and *PLCG1* as well as confirming associations of variants near *GCKR*, *ABCB11*, *LPL*, *HNF1A*, *FTO* and *SUGP1* with multiple phenotype domains [57]. A study in four Finnish cohorts encompassing 11,616 individuals identified a SNP, rs964184, near the *APOA1*/*C3*/*A4*/*A5* gene cluster on chromosome 11 as being associated with metabolic syndrome confirming a strong lipid gene component to the genetics of the metabolic syndrome [58]. Three GWAS studies have examined association of genetic variants in non-European populations. A study of 8842 Korean individuals identified two SNPs near *BUD13* as reaching genome-wide significance with a further eight SNPs reported to be of nominal significance including previously reported variants near *APOA5* and *LPL* [59]. Metabochip genotype array analysis of an African-American population identified 27 SNPs associated with the metabolic syndrome; however, only one (*APOC1*) was associated with all five components of the metabolic syndrome [60]. More recently, GWAS on a population of African ancestry identified variants near *RALYL*, *KSR2*, *MBNL1* and two variants specific to the African population near *CA10* and *CTNNA3* as being associated with metabolic syndrome [61].

While there is limited overlap between these GWAS, which may, at least in part, be due to slight differences in the definition of metabolic syndrome used, it is notable that many of the identified genes are associated with lipid traits. For example, variants near *ZNF259*, *APOB*, *LPL*, *APOA5*, *CETP* and *GCKR* have been previously shown to be associated with either low-density lipoprotein cholesterol, high-density lipoprotein cholesterol or triglycerides [62]. Overall, these studies indicate that while there may be a strong lipid element to the genetics underlying the metabolic syndrome, there is no clear evidence at this point in time of one or more pathways linking the different aspects of the syndrome. In a different approach to identify if common metabolic pathways link the various components of the metabolic syndrome, data from 1193 twin men from the Vietnam Era Twin Study of Aging with measures of adiposity (body mass index and waist circumference), blood pressure, insulin resistance (fasting insulin and glucose) and lipids (high-density lipoprotein cholesterol and triglycerides) were analysed [63]. This study suggested that adiposity may be the underlying factor which links the other factors in the metabolic syndrome with findings indicating shared genetic influences between insulin resistance, lipids and adiposity and also between blood pressure and adiposity.

## Low-Frequency and Rare Variants

Despite the increased success in identifying risk loci associated with insulin resistance and T2D, it has been estimated that these common variants account for only 25–44 % of the heritability of insulin resistance [64–66]. The contribution of low-frequency (MAF ≤ 5 %) and rare variants (MAF ≤ 0.5 %) to the heritability of T2D and the metabolic syndrome remains to be explored. Exome sequencing, where all protein-coding genes in the genome are sequenced to document all genetic variation in these regions, has recently been employed in an effort to identify novel low-frequency variants that may have large effects on common metabolic phenotypes. Exome sequencing in a Danish cohort of 2000 individuals with a follow-up in a further 15,989 individuals revealed associations of two common SNPs within *COBLL1* and *MACF1* with T2D and a low-frequency variant in *CD300LG* with fasting HDL-cholesterol [67]. While the biological function of the variants within *COBLL1* and *MACF1* is unknown, exploration of the functional effects of the risk variant rs72836561 at *CD300LG* revealed that this polymorphism is associated with reduced mRNA expression of *CD300LG* in skeletal muscle and adipose tissue, decreased insulin sensitivity and increased intramyocellular lipid content suggesting a link between this variant and aspects of the metabolic syndrome [68]. Exome sequencing in an Icelandic population revealed that a low-frequency variant in *CCND2* reduces the risk of developing T2D but, counterintuitively, is also associated with higher body mass index [69].

The 1000 Genomes Project was established to sequence, through a combination of both genome and exome sequencing, the genomes of 1092 individuals to aid identification of low-frequency and rare genetic variants across populations [70]. These reference panels, containing 38 million SNPs, have been used by the European Network for Genetic and Genomic Epidemiology (ENGAGE) consortium to successfully impute the genotypes of low-frequency variants and perform 22 GWAS for BMI, waist-hip ratio, fasting glucose and fasting insulin. A meta-analysis of these imputed GWAS identified two novel loci for BMI and two novel loci for fasting glucose, of which one was female-specific. The investigators also identified new lead SNPs at 29 established genetic loci including rs1260326 near *GCKR* for fasting insulin [71].

Another approach to identifying genetic variation associated with complex traits is to examine small populations which have been historically isolated. Such populations are often referred to as founder populations. Genotyping of 2733 individuals from the Greenlandic population using the Cardio-Metabochip followed by exome sequencing identified a common variant within *TBC1D4* as being associated with higher fasting glucose and reduced insulin sensitivity [72]. The effect of this p.Arg684Ter variant is to disrupt expression of the full-length TBC1D4 protein in skeletal muscle resulting in decreased insulin-stimulated glucose uptake.

Individuals with familial partial lipodystrophy appear predisposed to features of the metabolic syndrome and insulin resistance. Familial partial lipodystrophy tends to be associated with functional mutations in the *LMNA* (lamin A/C) gene [73] resulting in altered adipose tissue distribution. Genetic mutations, affecting either *LMNA* or the associated processing enzyme *ZMPSTE24*, have been shown to have a prevalence of 3 % in a small cohort of patients with metabolic syndrome [74] raising the possibility that mutations in the *LMNA* gene may contribute, or predispose, an affected individual to the metabolic syndrome.

Future approaches in identifying low-frequency and rare variants include the UK10K-cohort project which has combined whole-genome sequencing in a cohort of 3781 healthy individuals with exome sequencing of 6000 individuals with either rare disease, severe obesity or a neurodevelopmental disorder [75•]. This project has uncovered 24 million novel genetic variants and resulted in the generation of reference panels with increased coverage of low-frequency and rare variants to help facilitate the identification and contribution of rare variants in health and disease.

## Summary and Future Directions

Considerable recent progress has been made in the identification of genetic loci that are associated with insulin resistance and the metabolic syndrome. Some are directly involved in insulin action and glucose metabolism, while further work is required to determine the functional relationships between the genetic variants and insulin action. Nonetheless, the identification of validated variants has been challenging for a number of reasons. First, it is evident that the phenotypes (insulin resistance and metabolic syndrome) are influenced by lifestyle and environmental factors which need to be taken into account when exploring the underlying genetic architecture of these traits. Second, the insulin resistance phenotype itself is difficult to quantify, often relying upon indirect measures such as circulating insulin levels. Furthermore, the phenotype is often limited to whole body measurements with no comprehensive analysis of genetic variants influencing tissue-specific insulin resistance.

Despite these concerns, our appreciation of the genetic basis of insulin resistance and metabolic syndrome has evolved through a number of approaches described in this review. The challenge moving forward is to translate this knowledge into clinical practice to help predict and manage related conditions such as type 2 diabetes and cardiovascular disease.
